# Self-Assembly of Palmitic Acid in the Presence of Choline Hydroxide

**DOI:** 10.3390/molecules28227463

**Published:** 2023-11-07

**Authors:** Huifang Xu, Xin Liang, Song Lu, Meihua Gao, Sijia Wang, Yuanyuan Li

**Affiliations:** 1 College of Pharmacy, Henan University of Chinese Medicine, Zhengzhou 450046, China; xinliang_pharmacy@163.com (X.L.); sdlclusong@163.com (S.L.); sjwang13ecust@163.com (S.W.); liyy2018@hactcm.edu.cn (Y.L.); 2 School of Materials and Chemical Engineering, Xuzhou University of Technology, Xuzhou 221018, China

**Keywords:** palmitic acid, self-assembly, molar ratio, counter-ion, chain length

## Abstract

To disperse fatty acids in aqueous solution, choline, a quaternary ammonium ion, has been used recently. So far, only the self-assembly of myristic acid (MA) in the presence of choline hydroxide as a function of the molar ratio has been investigated, and, thus, the current understanding of these fatty acid systems is still limited. We investigated the self-assembly of palmitic acid (PA) in the presence of choline hydroxide (ChOH) as a function of the molar ratio (*R*) between ChOH and PA. The self-assemblies were characterized by phase contrast microscopy, cryo-TEM, small-angle X-ray scattering, and ^2^H NMR. The ionization state of PA was determined by pH, conductivity, and FT-IR measurements. With increase in *R*, various self-assembled structures, including vesicles, lamellar phase, rigid membranes (large sheets, tubules, cones, and polyhedrals), and micelles, form in the PA/ChOH system, different from those of the MA/ChOH system. The change in *R* induces pH variation and, consequently, a change in the PA ionization state, which, in turn, regulates the molecular interactions, including hydrogen bonding and electrostatic interaction, leading to various self-assemblies. Temperature is an important factor used to tune the self-assembly transitions. The fatty acid choline systems studied here potentially may be applicable in medicine, chemical engineering, and biotechnology.

## 1. Introduction

The current state of the Earth’s climate and the depletion of fossil fuels have resulted in a greater demand for the utilization of eco-friendly molecules. From the perspective of energy use, fatty acids, a class of carboxylic acids with an alkyl chain, offer an alternative to petroleum-derived surfactants. Fatty acids are a kind of cheap and valuable biosurfactant, which can be easily extracted from plant oils and animal fat tissues. The applications of fatty acids date back to thousands of years ago; at that time, our ancestors used soaps as detergents. In 1973, Gebicki and Hicks first reported the observation of oleic acid/sodium oleate vesicles facilitated by hydrogen bonding in aqueous solution [1]. Since then, more researchers have started to focus on the self-assembly behavior of saturated or unsaturated fatty acids in aqueous solutions as well as their applications in numerous fields [2,3,4,5,6,7,8,9,10,11]. Generally, fatty acids dispersed in alkaline aqueous solution at a specific temperature self-assemble into various aggregates, including micelles, vesicles, or lamellar phases, and oil droplets, depending on pH [2,3,5,10,11,12,13,14,15,16,17,18]. Different aggregates impart distinct properties to them, and they are extensively employed in fields such as food [19], medicine [9,20], cosmetics [21], chemical engineering [22,23], and primitive membrane models [8,10].

However, in practical applications, traditional sodium and potassium soaps of saturated long-chain fatty acids in aqueous solution have poor solubility at room temperature, showing a high Krafft point above 25 °C (e.g., ~60 °C for sodium palmitate [24]). To disperse fatty acids in water, three main strategies are commonly employed: chemical modification [25,26], mixing with cationic surfactants under their hydroxide form [27,28,29,30], and forming ion pairs with organic counter-ions [7,13,31,32,33,34,35,36]. Among them, chemical modification can enhance solubility by introducing hydrophilic groups into the molecular structure of fatty acid but this involves complex synthetic steps, which contradict the principle of green chemistry and sustainable development [27,28,29]. By mixing fatty acids with cationic surfactants at different molar ratios, for instance, myristic acid (MA) and cetyltrimethylammonium hydroxide, a variety of self-assemblies form in the salt-free systems including micelles, vesicles, nanodiscs, or icosahedra [27,28,30,31]. The last method for dispersing fatty acids effectively in an aqueous solution is by introducing large organic counter-ions (amines and cationic components) [31], where the solubility of fatty acids can be controlled by modulating the type of counter-ion [7,11,14,31,32,33,34,35,36,37,38]. For instance, Zana et al. [38] replaced sodium ion with tetraalkylammonium salt ion, a quaternary ammonium counter-ion, reducing the Krafft point of the stearic acid system from 70 °C to below 3 °C. The increase in solubility can be attributed to two primary factors, namely, by the hindrance of the regular crystalline arrangement by the bulky organic counter-ions (the main driving force) and by a weak binding between the organic counter-ions and the polar headgroups [24,39,40]. As for the dispersion systems of fatty acids in organic counter-ion under hydroxide form, also considered as salt-free catanionic systems, the molar ratio is one of the most important factors in the self-assembly process. Various self-assemblies, such as micelles, vesicles, lamellar phases, and facetted objects, form by changing the molar ratio between organic counter-ion and fatty acid [7,13,16,17,38].

In the past decades, choline, a quaternary ammonium ion of biological origin, has been used to disperse fatty acids [7,13,15,24,39,40]. Choline chemically refers to the cation (2-hydroxyethyl)trimethylammonium, which is also known as vitamin B4, an essential nutrient. This molecule is considered biocompatible and safe owing to the physiological degradability and natural decomposition derived from the hydroxyl group [24], which has attracted much attention for preparation of biocompatible deep eutectic solvents (DESs) [41] or ionic liquids (ILs) [42], combined with fatty acids. As a large ion of weak hydration and low surface charge density, choline is termed chaotropic, and is bound weakly to the alkyl carboxylates, preventing crystallization and promoting the formation of the self-assemblies [13,24,39,40]. Using the myristic acid/choline hydroxide system [7] as a model system, various self-assemblies can form by tuning the molar ratio: facetted objects (vesicles and discs), lamellar phases, and spherical micelles. However, the self-assembly of fatty acids in choline hydroxide solution may be affected by the alkyl chain length; therefore, it is important to study the self-assembly properties of other fatty acids with different chain lengths, such as palmitic acid with a carbon number of 16. Palmitic acid is the most common saturated fatty acid accounting for a significant portion (20–30%) of total fatty acids in the human body [43], which also stimulates us to study further from the perspective of surfactant self-assembly.

**Figure 1:**
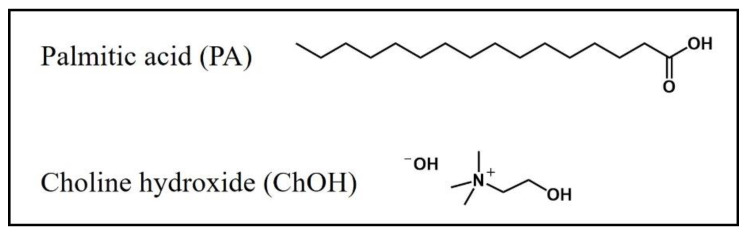
Molecular structures of palmitic acid and choline hydroxide.

In the present work, we investigated the self-assembly behavior of palmitic acid (PA) in the presence of choline hydroxide (ChOH) as a function of the molar ratio *R* (*R* = *n*_ChOH_/*n*_PA_) and temperature (*T*) (Figure 1). Our main purpose is to reveal the respective effects of the slight change in chain length of fatty acids on their self-assembly behaviors. Similar to the MA/ChOH system [7], an *R*-dependent self-assembly behavior was observed, but rigid membranes (large sheets, tubules, cones, and polyhedral structures) only form in the PA/ChOH system, different from those of the MA/ChOH system. In addition, temperature serves as another factor used to adjust the self-assembly transitions within this system. To our knowledge, this is the first report on the self-assembly behavior of the PA/ChOH system. The fatty acid choline systems studied here potentially may be applicable in medicine, chemical engineering, and biotechnology.

## 2. Results and Discussion

### 2.1. Effect of Molar Ratio R at the Macroscopic Scale: Phase Behavior

**Figure 2:**
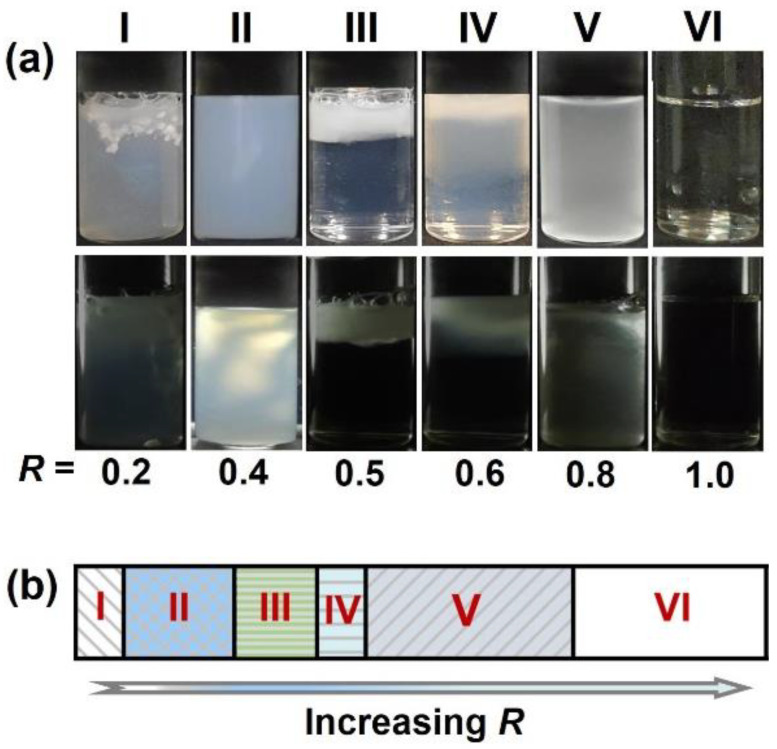
(**a**) Typical photographs of PA/ChOH solutions at 25 °C without (**up**) and with (**below**) crossed polarizers. (**b**) Phase regions divided for PA/ChOH system based on the molar ratio *R*. *C*_PA_ = 50 mM. Region I: turbid dispersion with precipitates; Region II: homogeneous turbid phase; Region III: phase separation with a turbid upper phase and a limpid lower phase; Region IV: phase separation with a slightly grayish gel-like phase between a turbid upper phase and a limpid lower phase; Region V: slightly grayish gel-like phase; and Region VI: homogeneous transparent limpid phase.

PA cannot dissolve in water, but it disperses easily in ChOH alkaline aqueous solution to give PA/soap or PA/ChOH mixtures. In this work, unless mentioned, the PA concentration (*C*_PA_) was fixed at 50 mM to focus on the dilute regime. The molar ratio, denoted as *R* (*R* = *n*_ChOH_/*n*_PA_), represents the ratio between ChOH and PA. The phase states were determined based on the appearance of the sample with and without crossed polarizers, and photographs of the typical PA/ChOH solutions are shown in Figure 2a. By simply increasing *R*, the PA/ChOH system exhibited an *R*-dependent phase behavior, and regions I to VI were designated accordingly (Figure 2b).

In region I, within a low *R* value (0.20 ≤ *R* ≤ 0.33 mM), precipitates were observed in the turbid dispersion without birefringence, showing that the amount of ChOH was not sufficient to solubilize the fatty acid. As *C* increases to 0.33 < *R* ≤ 0.44, corresponding to Region II, a homogeneous turbid bluish phase with birefringence was observed. The birefringence suggests the formation of lamellar structures in the solutions. In region III (0.44 < *R* ≤ 0.56), a slow macroscopic phase separation occurred, forming an aqueous two-phase system with a clear interface, in which the upper phase was turbid and milky without birefringence and the lower phase was transparent and limpid between crossed polarizers. With increase in *R* to in the range of 0.56 < *R* ≤ 0.68 (Region IV), the interface of the aqueous two-phase stage became blurry, and a slightly grayish gel-like phase with weak birefringence appeared between the upper turbid milky phase and the lower transparent phase. The length of gel-like phase increased with increase in *R*, accompanied by a decrease in that of the upper milky phase. With increase in *R* to the range of 0.68–0.91 (Region V), the upper milky phase vanished, and only the slightly grayish gel-like phase with weak birefringence was suspended in the system. The gel-like phase was turbid and slightly grayish aqueous suspension. In addition, the aqueous suspensions have a hydrogel behavior which may be induced by entanglements between aggregates [11], since they are more viscous qualitatively than micelle solutions (Region VI) but can still flow. We consider that the phase state in Region V was in the intermediate of solid state and gel state, so we call it a gel-like phase, which is similar to the case of aqueous binary mixtures of stearic acid and 12-hydroxystearic acid in excess ethanolamine [11]. At higher *R* (0.91 < *R* ≤ 2.0), the system transformed into a homogeneous transparent limpid single-phase stage.

Macroscopically, with increase in *R*, both the PA/ChOH system and the myristic acid (MA)/ChOH system [7] at 25 °C exhibit rich phase behaviors including turbid dispersion with precipitates, homogeneous turbid phase, aqueous two-phase stage, and homogeneous transparent phase. However, a slightly grayish gel-like phase was only observed between the regimes of the aqueous two-phase stage and homogeneous transparent phase in the PA/ChOH system while not in the MA/ChOH system reported [7]. This inspired us to further determine the aggregate structures of the PA/ChOH system at the microscopic scale.

### 2.2. Effect of Molar Ratio R at the Microscopic Scale: Structures of Aggregates

Typical sample solutions for the PA/ChOH system were chosen to characterize the microstructures formed in varied regions using phase contrast microscopy and cryo-TEM along with ^2^H NMR and SAXS techniques.

#### 2.2.1. Microscopy Observation

**Figure 3:**
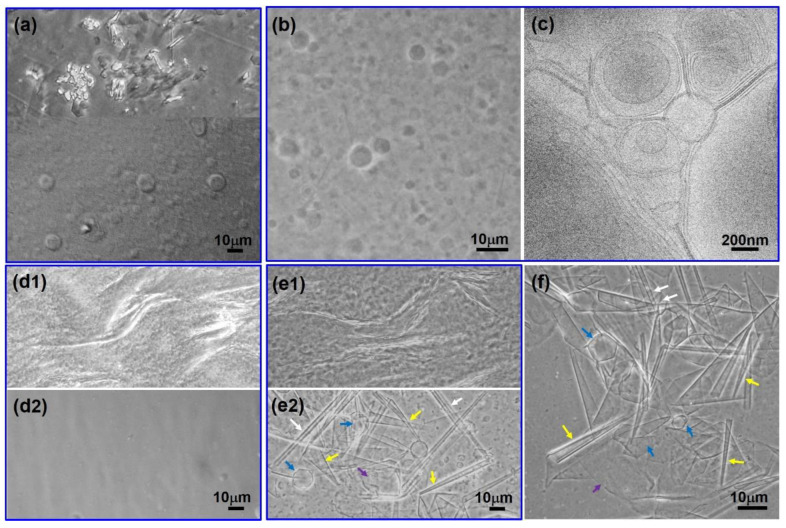
Phase contrast images (**a**,**b**,**d1**,**d2**,**e1**,**e2**,**f**) and cryo-TEM image (**c**) for PA/ChOH solutions at different *R*. (**a**) *R* = 0.2 in Region I, (**b**,**c**) *R* = 0.4 in Region II, (**d1**) *R* = 0.5 in Region III, upper phase, (**d2**) *R* = 0.5 in Region III, lower phase, (**e1**) *R* = 0.6 in Region IV, upper phase, (**e2**) *R* = 0.6 in Region IV, gel-like phase, and (**f**) *R* = 0.8 in Region V. The white, blue, yellow, and purple arrows refer to tubles, polyhedral structures, cones, and large sheet structures, respectively.

Figure 3 shows the phase contrast microscopy or cryo-TEM images for *R* = 0.2 (a), *R* = 0.4 (b, c), *R* = 0.5 (d1, d2), *R* = 0.6 (e1, e2), and *R* = 0.8 (f) at 25 °C. For R = 0.2 (Region I), precipitates occur in the turbid dispersion. As shown in Figure 3a, phase contrast microscopy revealed that both irregular precipitate particles (upper image) and polydisperse vesicles (lower image) coexist in the turbid suspension.

With *R* increasing to 0.4 in Region II, micron-size vesicles and lamellar bilayers (Figure 3b) were observed using a phase contrast microscope. As shown in Figure 3c, cryo-TEM of high-resolution further showed that the vesicles were polydisperse in size ranging from 100 nm to a few microns and most of the vesicles were of multi-lamellar structures. Meanwhile, stacked lamellar bilayers were clearly observed to coexist with the vesicles.

As *R* increased to 0.5 in Region III, the system separated into two phases. Figure 3(d1) showed that the aggregates in the turbid upper phase were stacked lamellar structures. No large aggregates were observed in the transparent lower phase (Figure 3(d2)), indicating the formation of small micelles [7,13,18] above critical micelle concentration [24], which is consistent with the limpidity of the solution. The existence of micelles was further detected by DLS (Figure S1), showing that micelle aggregates formed in the lower phase with ca. 3.8 nm in size, which is exactly twice the length of the fully extended PA molecule (~1.9 nm [44]).

With increase in *R* to 0.6 in Region IV, a slightly grayish gel-like phase formed between two phases. For the gel-like phase, various rigid membranes or crystallites (Figure 3(e2)) were observed, including large sheet structures (purple arrow in Figure 3(e2)), tubules (white arrow in Figure 3(e2)), cones (yellow arrow in Figure 3(e2)), and polyhedral structures (blue arrow in Figure 3(e2)). In addition, these rigid membranes exhibit polymorphism in many aspects. The tubules vary in diameter and length from place to place; the cones vary in morphological size and apex angle; the bilayer sheets and polyhedral structures also have varying curvatures or morphologies. For the turbid upper phase, the stacked lamellar phase still existed. The lower limpid phase was a clear solution where small-sized micelles formed as examined by phase contrast microscope and DLS (Figure S2).

With increase in *R* to 0.8 in Region V (Figure 3f), the microstructures in the grayish gel-like phase were rigid membrane structures (large sheet structures, tubules, cones, and polyhedral structures) (Figure 3(e2)), similar to the case in Region IV.

At higher *R* (*R* = 1.0, Region VI), PA molecules were almost completely deprotonated. The electrostatic repulsion between ionized PA molecules increased, leading to the formation of pure micelles evidenced by the phase contrast microscope and DLS (Figure S3).

#### 2.2.2. NMR Analysis

**Figure 4:**
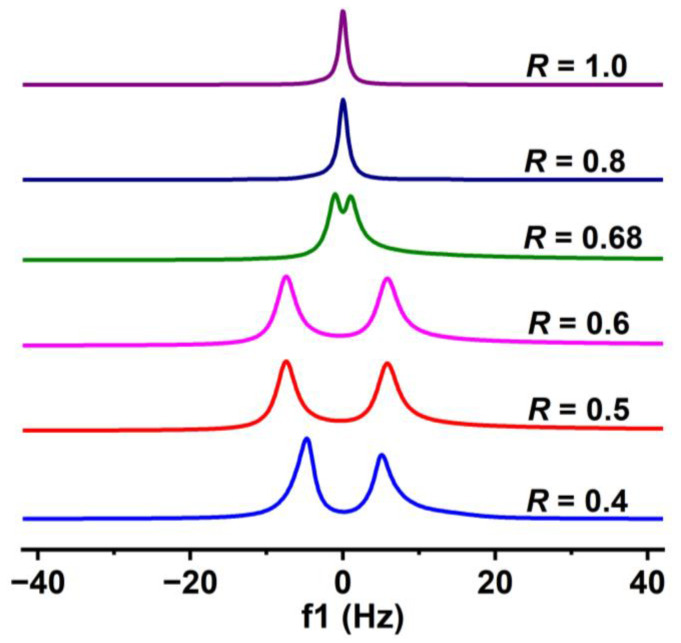
^2^H NMR spectra of the PA/ChOH systems as a function of *R* at 25 °C.

^2^H NMR has been widely used to detect the microstructure of aggregates [45,46,47,48]. ^2^H NMR line shapes are dominated by the interaction of the deuteron quadrupole moment with the electric field gradients at the nucleus [49]. In general, an isotropic phase exhibits a sharp singlet, while an anisotropic phase exhibits a doublet. The bilayer structures with certain periodicity are anisotropic macroscopically, so they exhibit a doublet in ^2^H NMR spectra. Figure 4 shows the ^2^H NMR spectra of typical deuterated aqueous solutions of the PA/ChOH system at 25 °C. With an increase in *R* from 0.4 to 1.0, the ^2^H NMR spectra show a change from a completely split doublet to a sharp singlet via an incompletely split doublet, which indicates an *R*-driven structural transition occurring [48]. In the *R* range of 0.4–0.6 (Regions II, III, and IV), a completely split doublet was observed, suggesting the presence of stacked bilayer structures [48]. This is consistent with the above microscopic results: stacked bilayer structures (Figure 3(c,d1,e1)) are always present in this *R* range. For the sample with *R* = 0.68, located at the boundary of Regions IV and V, the incompletely split doublet peak indicates the presence of the bilayer structures, but the arrangement of bilayers is not highly ordered. At *R* = 0.8 (Region V), a sharp singlet was observed, corresponding to the gel-like phase dispersed in the bulk phase. As shown in Figure 3f, the gel-like phase comprises various rigid membrane structures but lacks sufficient periodicity of lamellar structures. With increase in *R* to 1.0, the singlet indicates the formation of a homogeneous micelle solution. Therefore, the ^2^H NMR results further validate the microscopic observations: with an increase in *R*, vesicles, and lamellar phase, rigid membranes, and micelles form successively.

#### 2.2.3. SAXS Analysis

**Figure 5:**
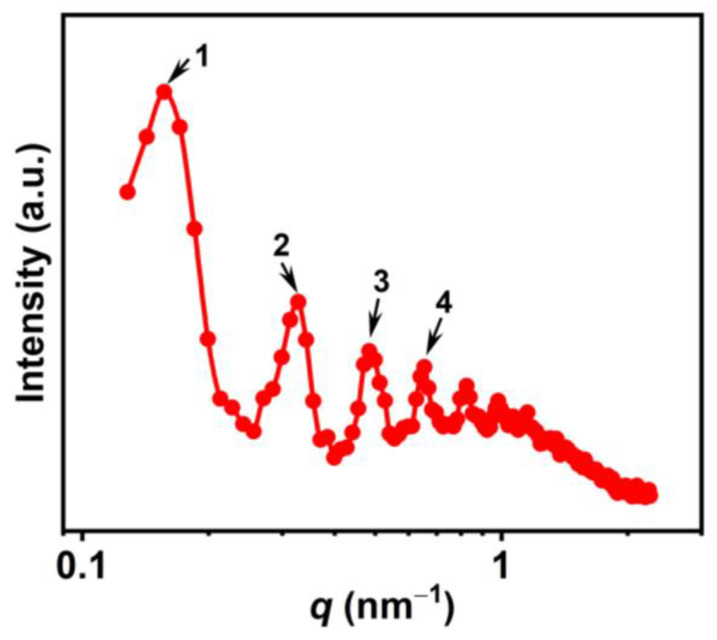
SAXS pattern for the upper phase at R = 0.5 in Region III of the PA/ChOH system at 25 °C. The numbers 1–4 represent four periodic scattering peaks.

The lamellar phase observed was analyzed using SAXS. Figure 5 shows the SAXS pattern for the upper phase at *R* = 0.5 in Region III of the PA/ChOH system at 25 °C, where *q* is the scattering factor, defined as *q* = (4*π*/*λ*)sin(*θ*/2), with *λ* being the wavelength of X-ray and *θ* being the scattering angle. For the upper phase at *R* = 0.5, in the low-*q* region, four periodic scattering peaks were observed at *q*_1_ (0.16 nm^−1^), *q*_2_ (0.32 nm^−1^), *q*_3_ (0.48 nm^−1^), and *q*_4_ (0.65 nm^−1^). The *q*_1_:*q*_2_:*q_3_*:*q*_4_ ratio closes to 1:2:3:4, corresponding to the Bragg scattering of stacked lamellar structures [50], which is consistent with the microscopic results (Figure 3(d1)) and ^2^H NMR results (Figure 4). Derived from the Bragg equation [12], *d* = 2π/*q*_1_, the *d*-spacing (layer spacing) for the lamellar phase was estimated from the first peak position to be ~39.25 nm. The *d*-spacing is larger than the twice fully extended length (~1.9 nm [44]) of the PA molecule, indicating the presence of large amounts of water between the lamellar bilayers. For the gel-like phase at *R* = 0.8 (Region V), periodic scattering peaks disappear (Figure S4), corresponding to the rigid membranes without sufficient periodicity.

### 2.3. Effect of the Molar Ratio R at the Molecular Scale: Ionization State

The ionization state of palmitic acid is a key parameter controlling hydrogen bonding and electrostatic interactions in various self-assembled structures [3,31]. With increase in *R*, the pH of the system changes, and, consequently, the ionization state of palmitic acid changes. To explore the intrinsic reason for the structural transition at the molecular scale, we investigate the influence of the molar ratio *R* on the ionization state by conducting pH and conductivity measurements along with FT-IR experiments.

**Figure 6:**
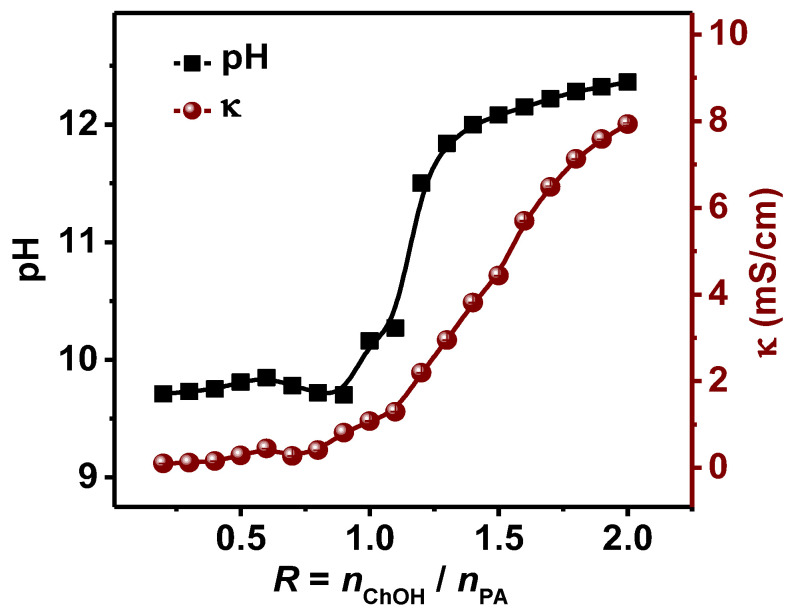
Variation of pH and conductivity for the PA/ChOH system as a function of *R* at 25 °C.

The pH and conductivity for the PA/ChOH system were obtained at different *R* at 25 °C. As shown in Figure 6, at low molar ratios (0.2 ≤ *R* < 0.9), the pH value remained around 9.7 ± 0.2, indicating that almost all OH^−^ ions added were consumed through acid-base reactions with PA molecules, and, thus, contributed little to increase in pH. Herein, three types of PA species coexist, i.e., non-ionized (neutral acid, RCOOH), ionized (deprotonated carboxylate soap, RCOO^−^) species, as well as the “acid-soap” dimers formed through hydrogen bonding between the two species. Similar to most fatty acid systems, it is the dimers that lead to the formation of various bilayer structures [18,51,52]. As *R* increased, the ionization degree (or relative content of RCOO^−^) increased, and the contents of RCOOH decreased. With increasing *R* above 1.0, the pH increased sharply. The PA molecules were fully ionized, and OH^−^ ions could no longer react with the non-ionized PA. Finally, the pH no longer changed and remained at around 12.

The corresponding conductivity results obtained were also shown in Figure 6. The conductivity remained very low at a low *R* ratio (0.2 ≤ *R* < 0.9). This can be ascribed to the reason that both OH^−^ and choline (Ch^+^) ions, which interact with the carboxylic or carboxylate groups were restricted by large vesicles and bilayer structures formed. From equimolarity (*R* = 1.0), the fatty acids were ionized completely, and the addition of ChOH in excess directly increased the conductivity of the solution, resulting in a dramatic rise in conductivity.

**Figure 7:**
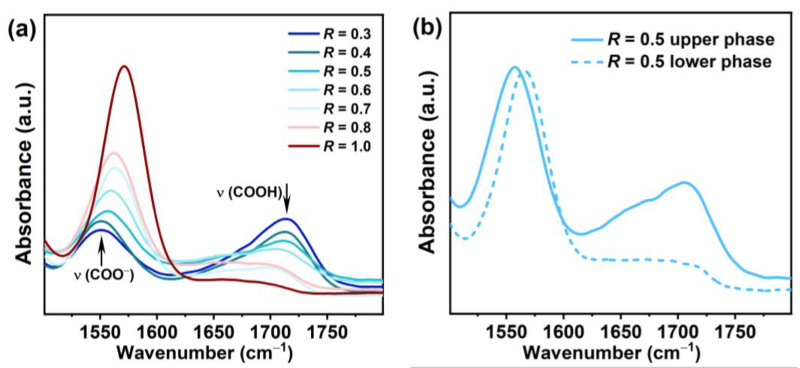
(**a**) FT-IR spectra for the PA/ChOH system with different *R*. (**b**) FT-IR spectra for the upper phase and lower phase at *R* = 0.5 in Region III.

Typical FT-IR spectra as a function of *R* were shown in Figure 7a. We focused on two specific peaks at around 1550 cm^−1^ and 1713 cm^−1^, which correspond to the characteristic adsorption bands of COO^−^ and COOH, respectively. In the range of 0.3–0.9, both peaks were observed, indicating that deprotonated RCOO^−^ and pronated RCOOH species coexisted in the systems. In addition, with increase in *R*, the intensity of the peak at 1550 cm^−1^ increased while the intensity of the peak at 1713 cm^−1^ decreased. This can be explained by the change in the ionization state. As *R* increased, the ionization degree increased, i.e., the amount of ionized RCOO^−^ species increased and that of non-ionized RCOOH species decreased. Furthermore, with the increase in *R*, the two peaks were slightly shifted from 1550 cm^−1^ to 1570 cm^−1^ and from 1713 cm^−1^ to 1700 cm^−1^ for the characteristic bands of COO^−^ and COOH, respectively, which can be attributed to the formation of hydrogen bonds [7]. At *R* = 1.0, only a peak at 1570 cm^−1^ was observed, indicating the complete deprotonation of PA, consistent with the pH and conductivity results.

It is worth noting that the spectra for the phase-separated samples (*R* = 0.5–0.8) were obtained about 30 min after equilibration at 25 °C before the phase separation occurred. To obtain information on the ionization state of the two separated phases, FT-IR measurements for the upper phase and lower phase at *R* = 0.5 in Region III after equilibration at 25 °C were conducted (Figure 7b). In the turbid upper phase, two absorption peaks were observed at 1556 cm^−1^ and 1705 cm^−1^; in the lower limpid phase, only a single absorption peak at 1565 cm^−1^ was observed. This indicates that the upper phase consisted of both the ionized and non-ionized species of PA molecules, while the lower phase only contained the non-ionized PA molecules.

Based on the results above, we establish the link between the phase behavior at the macroscopic level, the aggregate structures at the microscopic level, and the ionization state at the molecular level. By regulating *R*, the ionization state (i.e., the relative numbers of ionized and non-ionized species) changes, and, consequently, the intermolecular interactions (electrostatic interaction and hydrogen bonding) change, leading to the microstructure and phase transition.

### 2.4. Effect of Temperature

To investigate the temperature effect on this system, DSC and microscopy techniques along with the phase state observations were used to characterize the samples.

**Figure 8:**
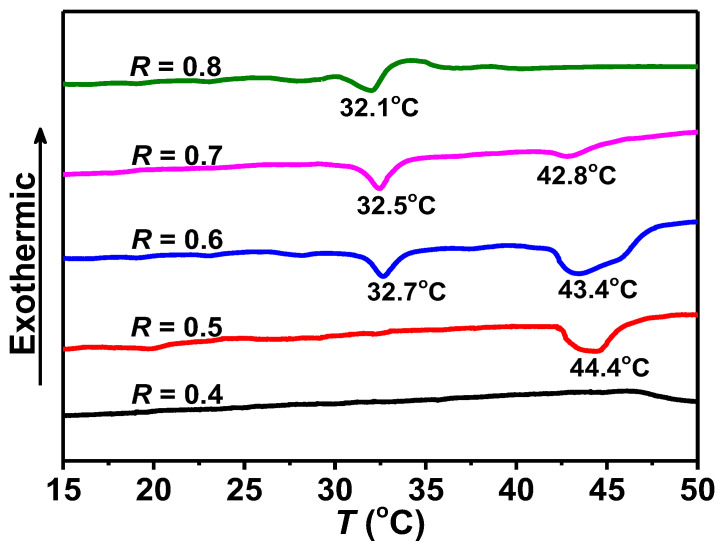
DSC traces for the PA/ChOH systems at different *R*.

As shown in Figure 8 and Figure S5, the DSC curves were obtained as a function of *R* in the range of 0.4–2.0 and *T* in the range of 15–50 °C. For *R* = 0.4, no peak can be observed in the range of 15–50 °C, suggesting that no phase transition occurred. For *R* = 0.5, a broad endothermic peak can be observed at around 44.4 °C. This may be attributed to the melting of the membranes [53,54] which can be correlated with the phase behavior and microscopy observations (discussed below).

For *R* = 0.6 and 0.7, one observes a complex endothermic behavior with a sharp peak (at around 32.7 °C and 32.5 °C for *R* = 0.6 and *R* = 0.7, respectively) and a broad one (at 43.4 °C and 42.8 °C for *R* = 0.6 and *R* = 0.7, respectively). As noted in the literature [14,53,54,55], the first peak possibly is often associated with the gel-to-fluid phase transition or chain-melting process; the second broad peak is related to the melting of the membranes, in which hydrogen bonds that remain within the fluid fatty acids are broken partially [56,57]. For *R* = 0.8, only a peak at around 32.1 °C was observed, corresponding to the gel-to-fluid phase transition temperature. By increasing *R*, the peaks were slightly shifted to lower temperatures, and completely vanished for *R* > 0.9 arising from the fact that the fatty acids were fluid in micelles (Figure S5).

**Figure 9:**
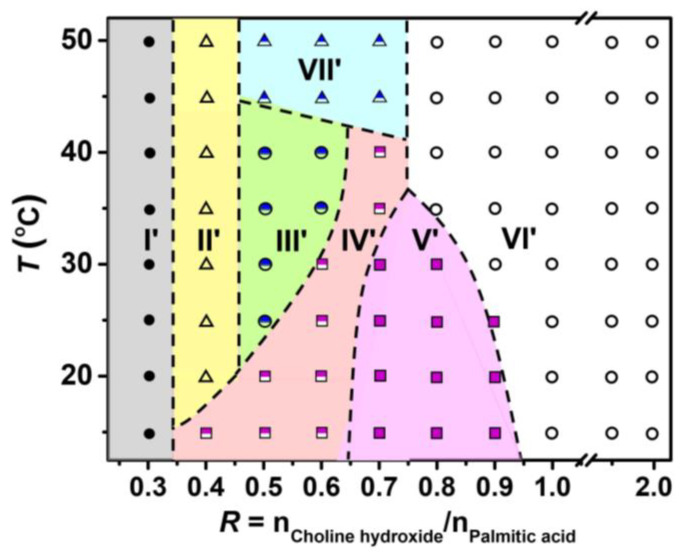
Phase diagram of the PA/ChOH system as a function of molar ratio *R* and *T*. *C*_PA_ = 50 mM. Region I′: turbid dispersion with precipitates; Region II′: homogeneous turbid phase; Region III′: phase separation with a turbid upper phase and a limpid lower phase; Region IV′: phase separation with a slightly grayish gel-like phase forming between a turbid upper phase and a limpid lower phase; Region V′: slightly grayish gel-like phase; Region VI′: homogeneous transparent limpid phase; and Region VII′: phase separation with a shorter turbid upper phase and a limpid lower phase.

To verify if the peaks determined by DSC correspond actually to the phase transition, macroscopic phase state observation and microscopy experiments were further performed as a function of *R* and *T*. The resulting phase diagram, depicting the relationship between *R*, T, and the phase state, was illustrated in Figure 9. The pictures for the samples within each region are shown in Figure S6. The phase diagram is divided into seven phase regions (I′–VII′). With fixing *T* at 25 °C, along the horizontal line, the phase states (I′–VI′) were consistent with the phase designation (I–VI) in Figure 2, respectively, in which turbid dispersion with precipitates, homogeneous turbid phase, aqueous two-phase stage, slightly grayish gel-like phase, and homogeneous transparent phase appear successively.

As shown in Figure 9, for 0.2 ≤ *R* ≤ 0.35 in Region I′, with increasing *T* from 15 to 50 °C, precipitates were observed in the turbid limpid dispersion. Microscopic observations for the samples at *R* = 0.3 and *T* = 15 °C and 50 °C (Figure S7) revealed the coexistence of vesicles and precipitate particles in this region.

**Figure 10:**
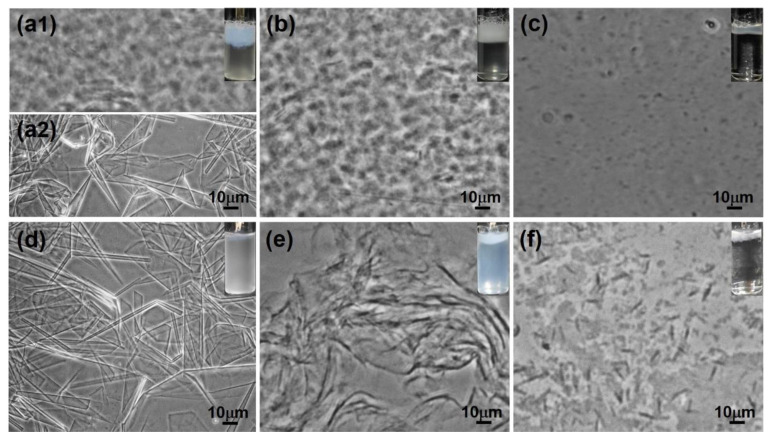
Phase contrast images for the PA/ChOH systems at *R* = 0.5 (**a1**,**a2**,**b**,**c**) and *R* = 0.7 (**d**–**f**). (**a1**,**b**,**c**,**e**,**f**) the upper phase; (**a2**) the lower phase. (**a1**,**a2**,**d**) *T* = 15 °C; (**b**,**e**) *T* = 35 °C; (**c**,**f**) *T* = 45 °C.

For *R* = 0.35–0.65 in the lower *T* regions (e.g., *T* = 15 °C for *R* = 0.4; 15 °C ≤ *T* ≤ 20 °C for *R* = 0.5; and 15 °C ≤ *T* ≤ 30 °C for *R* = 0.6), corresponding to Region IV′, the sample exhibited phase separation with a turbid and slightly birefringent upper phase and a lower limpid phase, which is similar with the phase state in Region IV (Figure 2) as noted above. The interface of the two phases was blurry, and a small amount of slightly grayish gel-like phase was observed to exist between these two phases. In this region, a stacked lamellar phase existed in the turbid upper phase, and rigid membranes of the gel-like phase suspended in the micellar bulk phase, as evidenced by Figure 10(a1,a2) for *R* = 0.5 and *T* = 15 °C.

At *R* = 0.4 and 15 °C < *T* ≤ 50 °C, the system always exhibited a homogeneous turbid state, showing that vesicles and bilayer structures exist in this region, as evidenced by the microscopy results in Figure 3c and Figure S8. At *R* = 0.5 and 0.6 (20 °C < *T* ≤ 40 °C), belonging to Region III′, the samples exhibited an aqueous two-phase state with a clear interface, with the upper lamellar phase and lower micelle phase as noted above. With increase in *T*, the volume of the upper phase became a little smaller (Figure S6c,d), indicating that the system entered into Region VII′. Further microscopy results (Figure 10b,c) for *R* = 0.5 showed that the large bilayer membranes in the upper phase melt into small-sized bilayer sheets with increasing *T* from 35 to 45 °C. At *R* = 0.7 and lower *T* of 15–30 °C (Region V′), a slightly grayish gel-like phase formed, and the microstructures herein were shown to be various rigid membranes by phase contrast image (Figure 10d). With increase in *T* to 30 °C < *T* ≤ 40 °C, the system entered into the phase-separated region IV′ again. The upper phase was determined to be flexible membrane structures (Figure 10e). With further increase in *T* to 40 °C < *T* ≤ 50 °C (Region VII′), the system exhibited phase-separation with a turbid upper phase and a clear lower phase. As shown in Figure 10f, small-sized bilayer sheets existed in the upper phase, similar to that of *R* = 0.5 and *T* = 45 °C (Figure 10c).

At higher *R* (0.75 < *T* ≤ 0.95), with increase in *T*, the grayish gel-like phase (Region V′) transformed into a homogeneous transparent single-phase stage (Region VI′), suggesting that the rigid membranes melted into micelles. For *R* > 0.95 corresponding to a high pH value, most of the PA molecules were ionized, contributing to the formation of micelles. In this region, with changing *T*, the samples kept a transparent single phase, because the alkyl chains of PA were in a fluid state in the whole *T* range.

The temperature is another factor affecting the self-assembly behavior in this system. During the chain-melting process, the alkyl chains changed from a rigid ordered gel state to a flexible disordered fluid state. We attempted to associate the DSC results with macroscopic phase states and microscopic observations. For instance, for *R* = 0.6 and 0.7, the first peak at about 32 °C corresponds to the melting of alkyl chains, which drives the transition from various rigid membranes (such as cones, polyhedral structures, tubules, and large sheet structures) in the systems to fluid membranes (Figure 10) [29,53,57]. Above the chain-melting temperature, the fluid bilayers were stabilized and maintained mainly due to the hydrogen bonds. Therefore, the second broad peak at about 43 °C corresponds to the breaking of the hydrogen bonds at the headgroup level [56,57]. As shown in Figure 10, with increasing *T* from 35 °C to 45 °C across the second peak, it seems that the fatty acid membranes melt partially, and only a small number of small-sized bilayer sheets existed in the upper phase (Figure 10). For *R* = 0.4 and 0.5, the number of the rigid membranes of the gel-like phase was too small to generate sufficient enthalpy change, and no peak was detected at about 32 °C. A broad peak at 44 °C was detected, owing to the melting of membranes, similar to the case of *R* = 0.6 and 0.7. For *R* = 0.8, only a gel-like phase can exhibit a phase transition from rigid membranes to soft micelles, corresponding basically to the peak at around 32 °C. At a high *R* above 0.9, no peak was observed, since fatty acids are always in a fluid state within micelles.

### 2.5. Self-Assembly Mechanism

**Figure 11:**
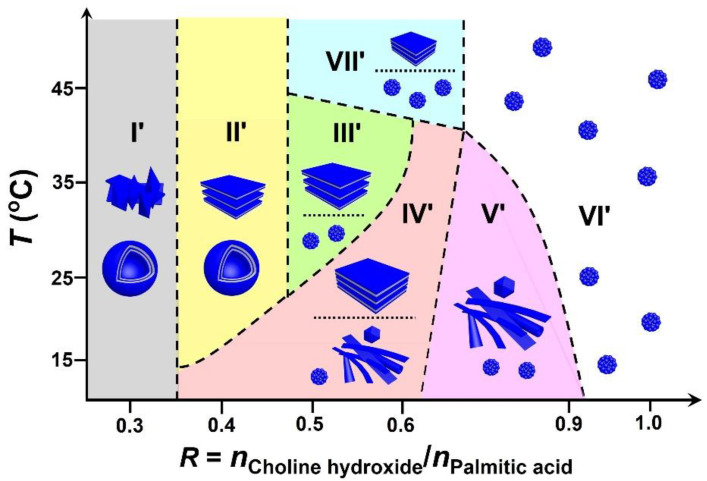
Schematic structural transformation as a function of *R* and *T*. Region I′: vesicles coexisting with precipitates; Region II′: vesicles and lamellar bilayers; Region III′: lamellar bilayers and micelles; Region IV′: lamellar bilayers and rigid membranes coexisting with micelles; Region V′: rigid membranes coexisting with micelles; Region VI′: micelles; Region VII′: smaller-sized bilayer sheets and micelles.

Our findings are summarized in Figure 11, illustrating the self-assemblies observed in the PA/ChOH system with changing *R* and *T*. It is known that the self-assembly morphology is determined by both weak interactions between molecules and also the packing parameter [58]. For fatty acid self-assemblies, hydrogen bonding and electrostatic interaction are the most important, depending on the ionization state of the molecules. According to the report by Israelachvili [59], the packing parameter is defined as *p* = *v*/*a*_0_*l*, where *a*_0_ is the cross-sectional area of the hydrophilic head group, and *v* and *l* are the volume and length of the hydrophobic chains in a fully extended state.

In our case, it is possible to induce the self-assembly transition by simply changing the molar ratio *R* between ChOH and PA. The molar ratio determines the pH of the solution, which, in turn, controls the ionization state of PA, leading to the change in intermolecular interactions. The value of *a*_0_ changes with the variation in the distance between the polar headgroups, which can be regulated by the intermolecular interactions. The packing parameter changes subsequently with the variation in *a*_0_. As shown in Figure 11, along the horizontal line of *T* = 25 °C, we first discussed the mechanism of self-assembly as a function of *R*.

In the low *R* range (0.20 ≤ *R* ≤ 0.33, Region I′), the pH value is around 9.5, and a small number of PA monomers were deprotonated to form ionized species. Three types of PA species, namely, non-ionized and ionized monomer species as well as the “acid-soap” dimers, coexist in the PA/ChOH system [18,60,61]. The molecular configuration of “acid-soap” dimers is similar to that of double-tailed amphiphiles with a higher *p* value (1/2 < *p* < 1), promoting the formation of vesicle or bilayer structures. Herein, the relative contents of the dimers and non-ionized species are higher than that of ionized species. The excess dimers and non-ionized species are more hydrophobic, leading to the formation of precipitates. Therefore, vesicles coexist with the precipitates (Figure 3a) in the system.

By increasing *R* (0.33 < *R* ≤ 0.44, Region II′), the pH increases very slowly, and remains around 9.7 ± 0.2, showing that the content of ionized species increases as well as the dimers. As a result, the number of bilayer assemblies (vesicles or lamellar bilayers) increases, and the precipitates vanished. Microscopic observations (Figure 3b,c) and ^2^H NMR analysis (Figure 4) confirm the coexistence of vesicles and bilayer structures. The threshold of *R* between the regimes containing bilayers coexisting with precipitates and only pure bilayers without precipitates is lower than that of MA/ChOH system [7], in which bilayers (facetted vesicles and discs) begin to form at about *R* = 0.4. This can be ascribed to a change of the packing parameter *p*. With increase in the length of hydrophobic alkyl chain from 12 to 14, the value of *v*/*l* increases [47,59], and the value of *a*_0_ remains constant with the same choline headgroup, resulting in an increase in *p*. Therefore, we can hypothesize that the change of packing parameter *p* facilitates the formation of bilayers at lower *R* compared with the MA/ChOH system.

In the intermediate range of *R* (0.44 < *R* ≤ 0.56, Region III′), the relative content of ionized species increases, leading to the enhancement of the electrostatic interaction between headgroups, which corresponds to a large *a*_0_ value and a concomitant low *p* value (*p* < 1/3), and micelles formed. In addition, the increase in dimers promotes the formation of the lamellar phase. Because of the density difference (*ρ*_PA_ < *ρ*_water_), a macroscopic phase separation occurs after equilibrating for a week with the PA-rich lamellar phase located in the upper phase and the PA-poor dilute micelle phase located in the lower phase. Similar phase separation also occurs in mixtures of myristic acid and organic amines [7,13], as well as lauric acid and inorganic bases such as cesium hydroxide [5].

With further increasing *R* (0.56 < *R* ≤ 0.68, Region IV′), the relative content of ionized species increases. In addition to the original lamellar phase and the micelle phase, an intermediate gel-like phase forms, which has been confirmed to consist of various rigid frozen membranes, including large sheet-like structures, tubules, cones, and polyhedral structures. The phenomenon is different from that of the common fatty acid/alkali system, in which insoluble particles (precipitates or oil droplets), bilayer structures (vesicles or lamellar phase), and micelles form, in turn, with increase in the amount of alkali. Note that the temperature herein is lower than the chain-melting temperature (~32 °C) obtained in Figure 8, and the bilayers are composed of crystallized fatty acids with alkyl chains in the gel state. The various rigid membranes lead to the formation of edges at the bilayer boundaries as in the case of nanodisks [27], bicelles [62], or regular hollow icosahedra [28], and hollow cones [32] in the literature. This can be attributed to the electrostatic repulsions between the polar heads of surfactants, because the bilayers are in an excess of anionic species [27,28,32,62]. In this *R* range, upon cooling from a clear solution at 75 °C in the sample preparation, a portion of the ionized PA species are expelled to form the edges of bilayers to minimize the bending energy, and the dimers and non-ionized species participate in forming the faces of the rigid membranes.

With increasing *R* to Region V′ (0.68 < *R* ≤ 0.91), the relative content of ionized species increases, and that of non-ionized species and dimers decreases, corresponding to the enhancement of electrostatic repulsions between head groups. This leads to the formation of micelles and various rigid membranes discussed above, along with the disappearance of the lamellar phase.

For *R* > 0.91, corresponding to a high pH above 10, almost all of the PA molecules were in their ionized state. In this case, the strong electrostatic repulsion between the ionized PA species leads to a large *a*_0_ value and a concomitant low *p* value (*p* < 1/3), and micelles form in solution, corresponding to the transparent single-phase solution.

In addition, temperature is an important factor to control the self-assembly transitions of the PA/ChOH system. The temperature affected the self-assembly due to the chain-melting process and disruption of the hydrogen bond at the headgroup level [56,57], similar to some other catanionic systems containing fatty acids and organic counter-ions [14,53,56,57].

## 3. Materials and Methods

### 3.1. Materials

All of the chemicals were used as received. Palmitic acid (≥99% purity) was purchased from Sigma-Aldrich, Shanghai, China. Choline hydroxide (47–50 wt% in water) was obtained from Tokyo Chemical Industry Co., Ltd., Shanghai, China. Ultrapure water (resistivity: 18.25 MΩ·cm) was obtained using a UPR-II-20T purification system (Sichuan ULUPURE Ultrapure Technology Co., Chengdu, China).

### 3.2. Sample Preparation and Phase State Observation

The designed quantity of palmitic acid was weighed into a sample vial, and choline hydroxide stock solution (1.65 mol/L) and ultrapure water were added to obtain a series of mixed solutions with different molar ratio *R* (*R* = *n*_ChOH_/*n*_PA_, where *n* is the molar concentration). To promote dissolution, the mixed solutions were heated at 75 °C for ~30 min and homogenized using a vortex mixer until the powders were completely dispersed. The resulting solutions were left to stand at a designed temperature under a N_2_ atmosphere for one week before use.

The phase state of the samples was determined by visual observation aided by two crossed polarizers.

### 3.3. Measurements

*Phase contrast microscopy.* An Olympus CKX31 inverted microscope (Guangzhou Mingmei Photoelectric Technology Co., Ltd., Guangzhou, China) with a phase contrast mode equipped with a ToupCam digital camera was used at 40× magnification to collect images. Each sample was thermostated using a TP-C110-MO-2 thermoplate (accuracy ± 0.1 °C ).

*Cryogenic transmission electron microscopy (cryo-TEM).* For cryo-TEM observation, the sample was prepared in a highly humid environment. A solution of 5 μL was loaded onto a carbon-coated copper grid. Then, the excess sample solution was removed by blotting the grid with two pieces of filter paper, leaving a thin liquid film on the grid mesh holes. After approximately 10 s, the grid was quickly transferred into liquid ethane that had been cooled by liquid nitrogen. The vitrified sample was then placed in a specific cryogenic specimen holder and examined using a JEM-1400 TEM (JEOL, Tokyo, Japan).

*Small-angle X-ray scattering (SAXS)*. SAXS measurements were conducted using a SAXSess diffractometer (Anton Paar, Graz, Austria) operating at 40 kV and 30 mA with a K_α_ radiation of 1.54 Å. The sample solution was carefully placed into a capillary tube, and the temperature was maintained at a constant level using an Anton Paar TCS 120 temperature control unit connected to the SAXSess instrument.

*Deuterium nuclear magnetic resonance (^2^H NMR)*. ^2^H NMR data were acquired on a Bruker Avance 500 spectrometer (Bruker Corp., Fällanden, Switzerland) that was equipped with a pulsed field gradient module (*z*-axis). The samples were prepared in 2 mL vials and homogenized by vortexing. Each spectrum was obtained by accumulating 64 scans, and a recycle delay of 1.0 s was employed.

*The pH and conductivity measurements*. The pH values of the solutions were determined using a FE28 pH meter (Mettler Toledo, Stockholm, Sweden) equipped with a LE422 glass micro-electrode. Conductivity measurements were conducted on a DSJ-308F digital conductivity meter (Shanghai REX Instrument Factory, Shanghai, China) with a DJS-1D glass electrode. Each reported value was the average of three separate measurements.

*Fourier transform infrared spectra (FT-IR)*. To obtain the FT-IR spectra for the samples, a Tensor II FT-IR spectrometer (Bruker Corp., Bremen, Germany) with an ATR accessory was positioned at the sample location of the instrument. The spectral resolution of the spectrometer was set to 4 cm^−1^.

*Differential scanning calorimetry (DSC)*. DSC traces were obtained on a Q2000 calorimeter (TA Instruments, New Castle, DE, USA) in a nitrogen atmosphere at a heating rate of 2 °C/min, within a temperature range of 15 to 70 °C.

## 4. Conclusions

In this work, we studied the self-assembly of PA in the presence of ChOH solution as a function of the molar ratio *R* and temperature. For this simple fatty acid system, various self-assembled structures, including vesicles, lamellar phase, rigid membranes, and micelles, form by changing *R*. Similar to the MA/ChOH system [7], an *R*-dependent self-assembly behavior was observed, but rigid membranes (large sheets, tubules, cones, and polyhedral structures) only form in the PA/ChOH system. The change in *R* induces pH variation, and, consequently, a change in the ionization state of PA, which, in turn, regulates the molecular interactions including hydrogen bonding and electrostatic interaction, promoting the formation of various self-assemblies. In addition, temperature is another factor used to tune the self-assembly transitions in this system. The temperature affected the self-assembly because of the gel/fluid transition of the alkyl chains or the disruption of the hydrogen bonding that remains within the fluid fatty acid bilayers above the chain-melting temperature. This work deepens the understanding of palmitic acid in the presence of choline hydroxide (as a quaternary ammonium counter-ion) and also provides valuable information for their practical applications as green surfactants, such as in medicine, chemical engineering, and biotechnology.

## Data Availability

The data presented in this study are available on request from the corresponding authors.

## Supplementary Materials

The following supporting information can be downloaded at: https://www.mdpi.com/article/10.3390/molecules28227463/s1, Figure S1: Size distribution of the lower phase of the PA/ChOH system at *R* = 0.5; Figure S2: (a) Phase contrast image and (b) size distribution of the lower phase of the PA/ChOH system at *R* = 0.6; Figure S3: (a) Phase contrast image and (b) size distribution of the PA/ChOH system at *R* = 1.0; Figure S4: SAXS pattern for the PA/ChOH system at R = 0.8 in Region V; Figure S5: DSC curve for the PA/ChOH system at *R* = 1.0; Figure S6: Photographs of PA/ChOH systems in water at different *T* with (top) and without (down) crossed polarizers. (a) *R* = 0.3, (b) *R* = 0.4, (c) *R* = 0.5, (d) *R* = 0.6, (e) *R* = 0.7, and (f) *R* = 0.8; Figure S7: Phase contrast images for the PA/ChOH systems at (a) *R* = 0.3, *T* = 15 °C and (b) *R* = 0.3, *T* = 50 °C; Figure S8: Phase contrast images for the PA/ChOH systems at *R* = 0.4. (a) *T* = 35 °C, (b) *T* = 50 °C.
