# Supplementary material for: Self-Assembly of Palmitic Acid in the Presence of Choline Hydroxide
Source: Molecules. 2023 Nov 7;28(22):7463. doi: 10.3390/molecules28227463 (PMC10673190; doi:10.3390/molecules28227463)
Supplement: Supplementary file 1 [file molecules-28-07463-s001.zip › molecules-2682472-supplementary.pdf]

Supporting Information

Self-assembly of palmitic acid in the presence of choline hydroxide

Huifang Xu [1,*], Xin Liang [1], Song Lu [1], Meihua Gao [2,*], Sijia Wang [1], Yuanyuan Li [1]

[1] College of Pharmacy, Henan University of Chinese Medicine, Zhengzhou 450046, P.R. China

[2] School of Materials and Chemical Engineering, Xuzhou University of Technology, Xuzhou 221018, P.R. China

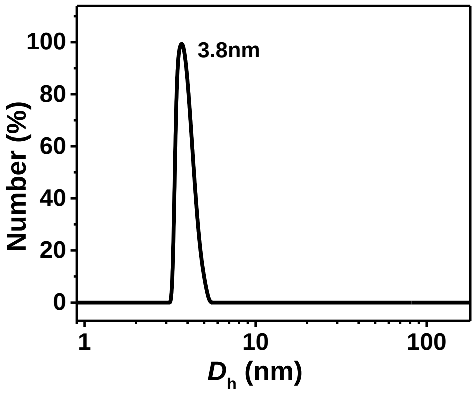

Figure S1. Size distribution of the lower phase of the PA/ChOH system at R = 0.5.

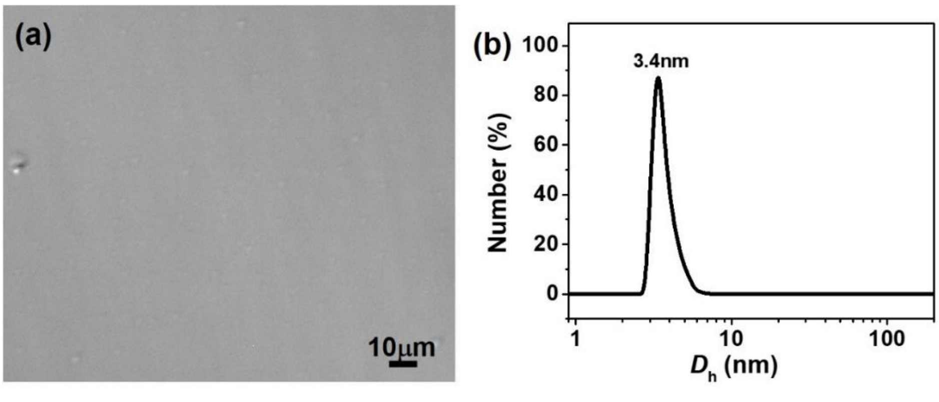

Figure S2. (a) Phase contrast image and (b) size distribution of the lower phase of the PA/ChOH system at R = 0.6.

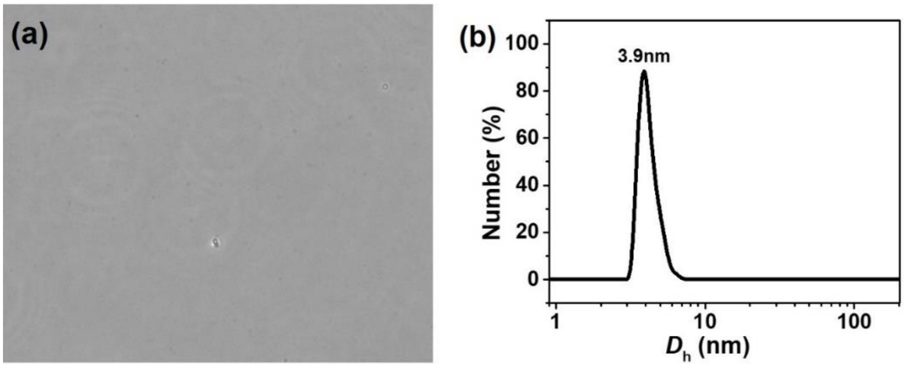

Figure S3. (a) Phase contrast image and (b) size distribution of the PA/ChOH system at R = 1.0.

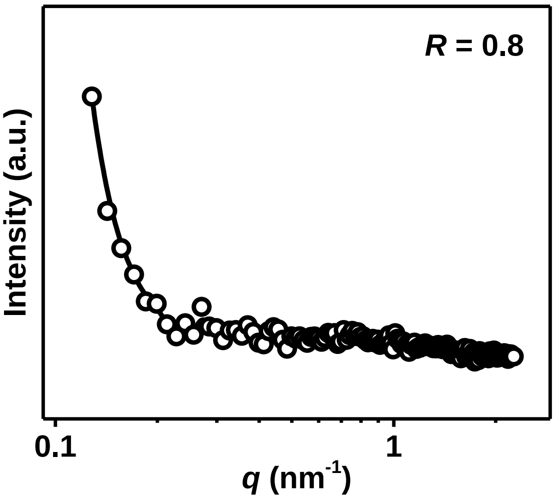

Figure S4. SAXS pattern for the PA/ChOH system at R = 0.8 in Region V.

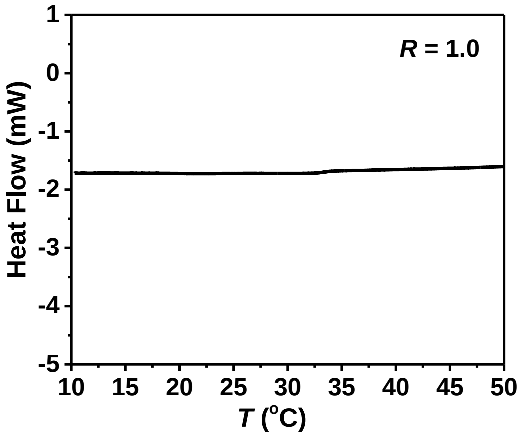

Figure S5. DSC curve for the PA/ChOH system at R = 1.0.

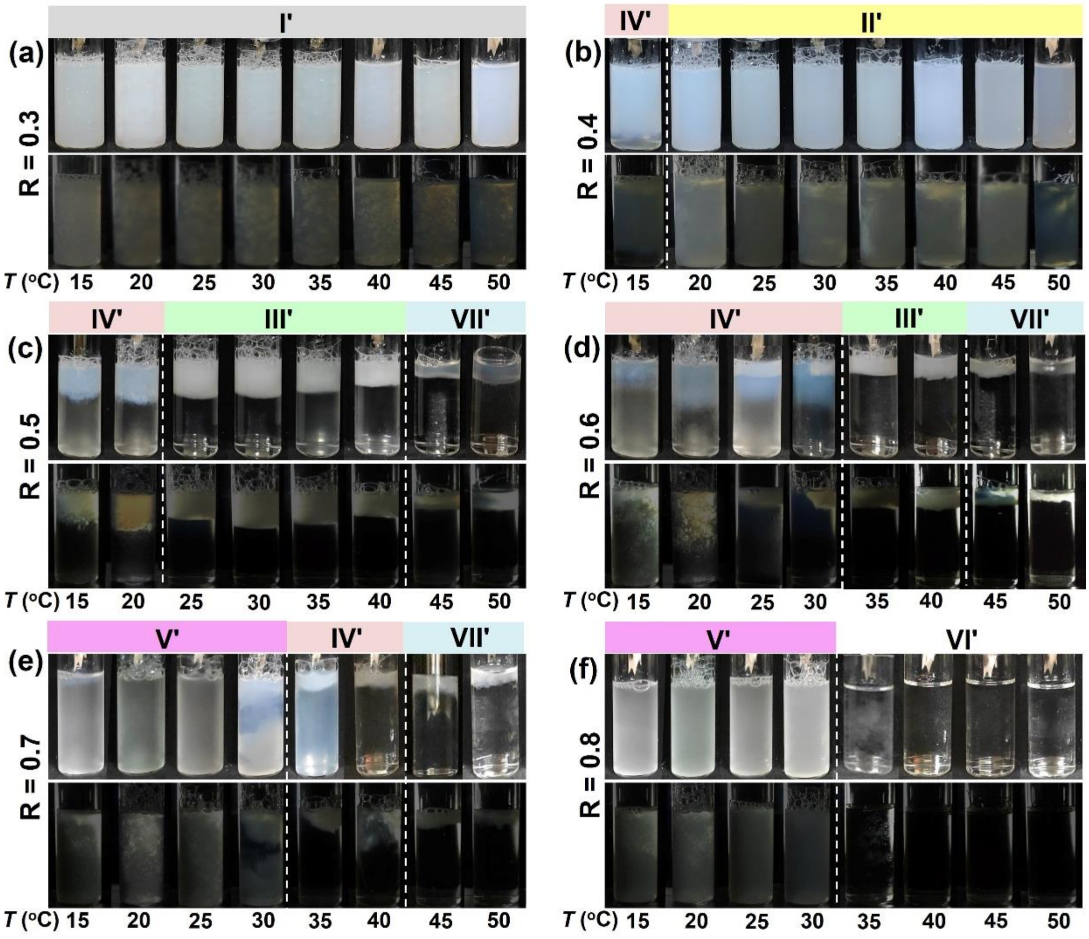

Figure S6. Photographs of PA/ChOH systems in water at different T with (top) and without (down) crossed polarizers. (a) R = 0.3, (b) R = 0.4, (c) R = 0.5, (d) R = 0.6, (e) R = 0.7, (f) R = 0.8.

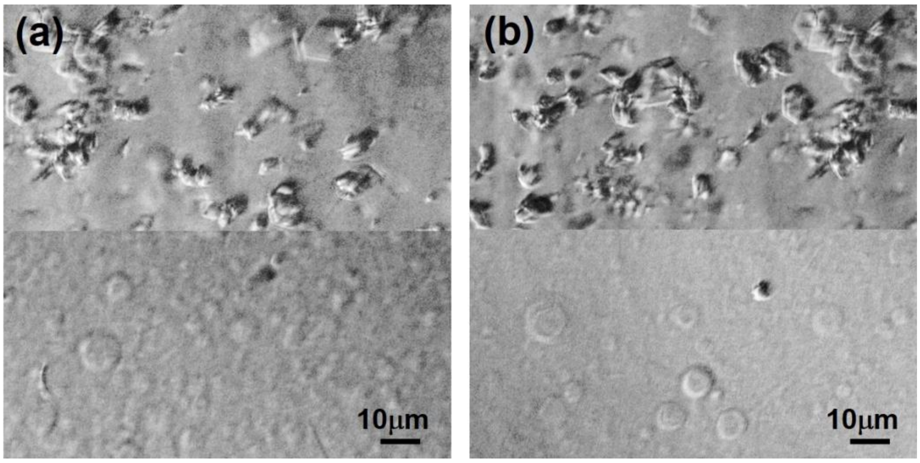

Figure S7. Phase contrast images for the PA/ChOH systems at (a) R = 0.3, T = 15 °C and (b) R = 0.3, T = 50 °C.

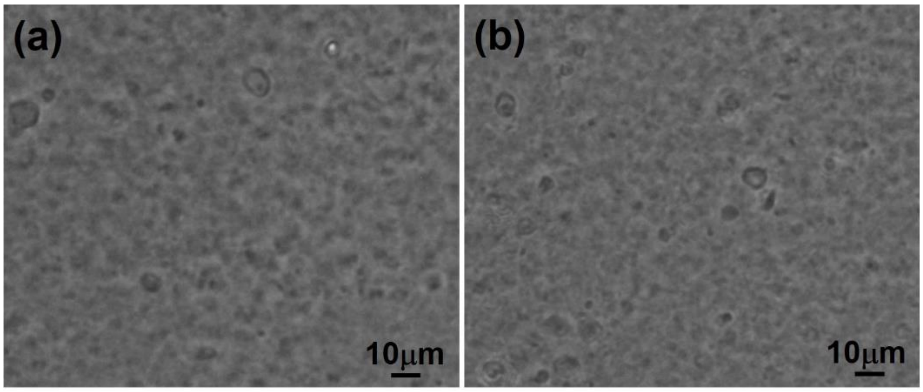

Figure S8. Phase contrast images for the PA/ChOH systems at R = 0.4. (a) T = 35 °C, (b) T = 50 °C.
